# Unique Nutcracker Phenomenon Involving the Right Renal Artery and Portal Venous System

**DOI:** 10.1155/2014/579061

**Published:** 2014-07-01

**Authors:** Maximilian Stephens, Sarah Kate Ryan, Roger Livsey

**Affiliations:** ^1^University of Queensland School of Medicine, Herston Road, Herston, Brisbane, QLD 4006, Australia; ^2^Department of Medical Imaging, Mater Misericordiae Hospital, Raymond Terrace, South Brisbane, QLD 4101, Australia

## Abstract

The nutcracker phenomenon is usually caused by compression of the left renal vein by the superior mesenteric artery anteriorly and the aorta posteriorly, although variations of this anatomy have previously been reported. We observed a nutcracker phenomenon in a 42-year-old female who underwent portal venous phase computed tomography of the body for oncologic workup. She had no documented proteinuria or hematuria. Multiplanar reconstructions demonstrated an enhancing left renal vein draining into the left ovarian vein without draining into the inferior vena cava due to external compression immediately before the renocaval junction. The left renal vein was compressed between the right renal artery and the portal vein. This type of nutcracker has not been previously reported in the literature and represents a new variation.

## 1. Introduction

The nutcracker phenomenon (NCP) and the nutcracker syndrome (NCS) have been recognized more frequently in the literature over the last few years. These reports describe external compression of the left renal vein (LRV), typically between the superior mesenteric artery (SMA) and the aorta, as was first described clinically by El-Sadr and Mina in 1950 [1]. This compression of the LRV restricts venous outflow and causes reciprocal dilatation of the hilar portion of the LRV and often the tributary left gonadal vein. NCP differs from NCS in that NCS is “symptomatic” or detectable with urinalysis, although no strict criteria exist as to the necessary extent of symptoms to define NCS [2]. NCS may manifest as macro- or microscopic hematuria, flank pain, proteinuria, varicocele, or orthostatic intolerance [2, 3]. Although compression of the LRV between the SMA and aorta is the most common scenario, variations to this have been reported:

- aorta and spinal column or “posterior” nutcracker phenomenon [4];
- arching left gonadal artery and psoas major muscle [5];
- SMA and right renal artery [6, 7];
- circumferential fibrous tissue [2, 8];
- retroperitoneal neoplasms/lymphadenopathy [2].

Here we describe external compression of the LRV by the right renal artery (RRA) and the portal vein (PV), which resulted in a nutcracker phenomenon with collateral outflow through the left ovarian vein.

## 2. Case Report

**Figure 1:**
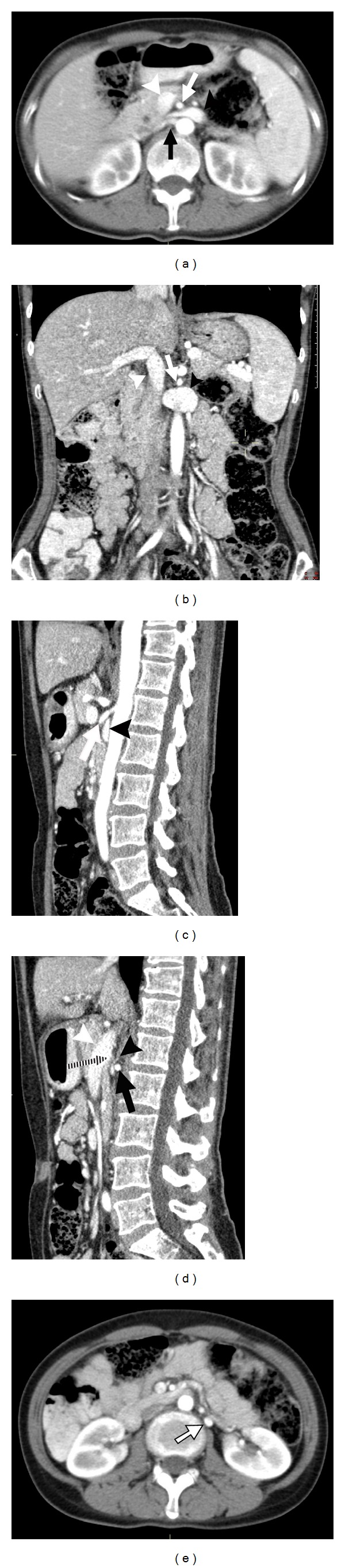
Multiplanar reconstruction of a nutcracker phenomenon. (a) Axial view: the left renal vein is compressed between the portal vein anteriorly and the right renal artery posteriorly. The left renal vein is patent after it traverses behind the superior mesenteric artery although minor compression occurs at this point. (b) Coronal view: the left renal vein ceases abruptly before the renocaval junction. (c) Sagittal view: the left renal vein is patent as it traverses behind the superior mesenteric artery. (d) Sagittal view: the left renal vein is compressed between the right renal artery and the portal vein. Adjacent retroperitoneal fat is present but not the offending structure. (e) Axial view: the left ovarian vein is dilated and enhancing, whilst the right ovarian vein is not appreciated. White arrowhead: portal vein; white arrow: superior mesenteric artery; black arrowhead: left renal vein; black arrow: right renal artery; white arrow with black border: left ovarian vein; black and white striped arrow: posterior bend of portal vein compressing left renal vein.

**Figure 2:**
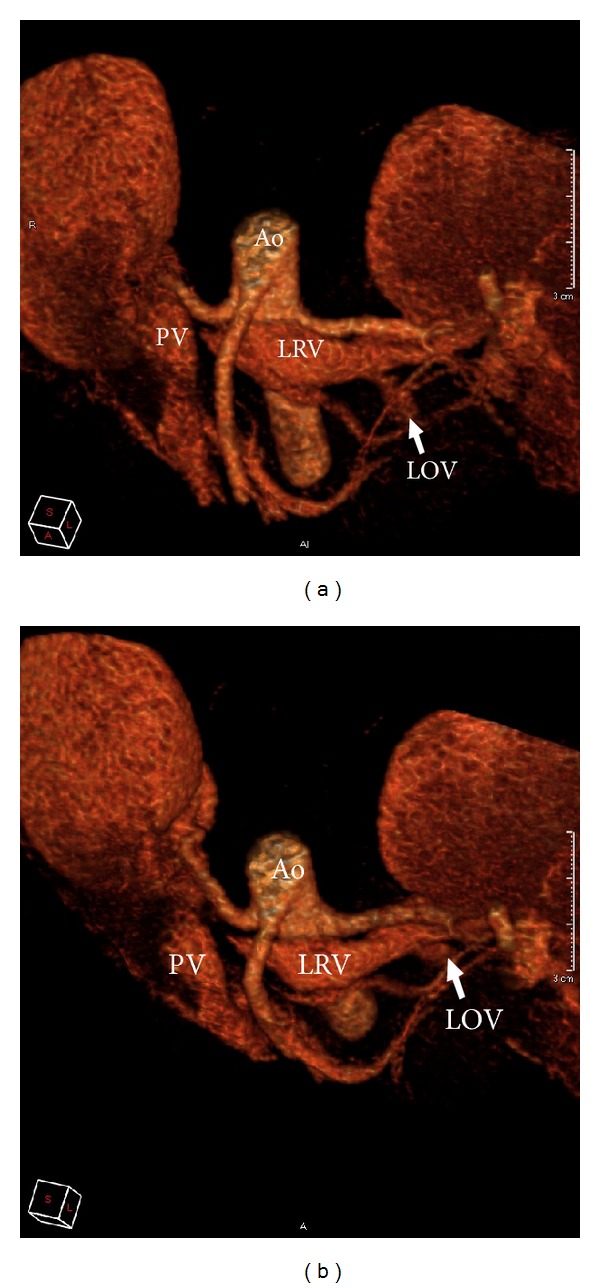
(a) and (b) 3D reconstructions of a nutcracker phenomenon. The left renal vein is compressed as it approaches the right renal artery and portal vein. Ao: aorta; PV: portal vein; LRV: left renal vein; LOV: left ovarian vein.

A 42-year-old Asian female presented with a 6-month history of left neck pain. This was on a background of left mastectomy for invasive ductal carcinoma 12 months prior, with postoperative chemoradiation. She also had chronic e-antigen negative hepatitis B but had unremarkable liver enzymes and hepatitis B DNA levels whilst on tenofovir. Examination was unremarkable except for postradiotherapy changes to the left chest wall. Portal venous phase computed tomography (CT) of the neck, chest, abdomen, and pelvis was performed (Philips Brilliance-64 MDCT, Philips Healthcare, Cleveland, OH, USA) to rule out metastatic disease. All studies were normal except for an observed nutcracker phenomenon (Figures 1 and 2).

The LRV appeared to be dilated, measuring 10.8 mm anteroposterior (AP) × 19.2 mm craniocaudal (CC) at its most dilated point, then tapering to complete occlusion, measuring 1.5 mm AP × 7.7 mm CC before the obstruction. This yielded a decrease in functional diameter by 86% and a cross-sectional proportion of 18 : 1. These measurements far exceed the suggested nutcracker phenomenon criteria of >50% and >4 : 1, respectively [2, 3]. The LRV appeared to be externally compressed anteriorly by the PV and posteriorly by the RRA immediately after its takeoff from the aorta. Compression of the LRV occurred proximally to the renocaval junction. Contrast filled the entire LRV; however, no contrast was seen in the inferior vena cava (IVC) (Figure 1). Of note, the right renal vein was patent with contrast seen to reach the right renocaval ostium; however, this occurred in a minute amount, inferior to the LRV, leaving the IVC predominantly unenhanced. Contrast was also seen to enhance the left ovarian vein (LOV) from the LRV to the level of the left ovary whilst the right ovarian vein did not enhance. The LOV appeared dilated, measuring 6.7 mm AP × 5.9 mm transverse at its most dilated point, although its caliber did not change significantly throughout its course. Blood analysis performed 3 weeks prior showed a creatinine of 58 *μ*mol/L (reference range 64–108 *μ*mol/L); however, no urinalysis was performed.

## 3. Discussion

The exact prevalence of NCS or NCP is unknown, although one series found evidence of asymptomatic “renal venous distension” in 72% of abdominal CTs [10]. There appears to be a slight predilection for females in their second to fourth decades of life [2, 8]. Although there is no concrete delineation between NCS and NCP, it is generally accepted that NCS is simply NCP with classical symptoms or evidence of renal damage.

**Figure 3:**
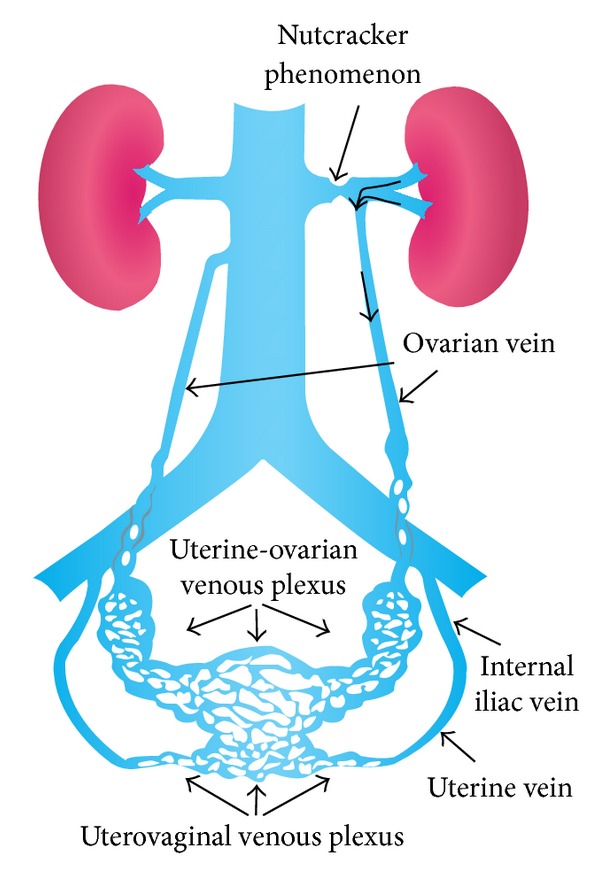
Anatomic illustration demonstrating the key venous anastomoses that facilitate shunting of renal blood when a nutcracker scenario arises. Image adapted with permission from Umeoka et al., Radiographics, 2004, 24 : 197, RSNA [9].

Our patient matched the classic demographic, yet was asymptomatic, and had a normal blood creatinine, favoring NCP over NCS. A urinalysis would have added considerably to the clinical picture to rule out proteinuria and hematuria. Although there was no evidence of decompensation by the left kidney, there were radiological findings that indicated significant venous shunting had been occurring through the left ovarian vein. The lack of contrast filling of the IVC, despite a contrast-laden LRV, in conjunction with a dilated and enhancing full length LOV suggests that venous blood from the LRV was shunted by collateral outflow through the LOV due to obstruction of the LRV. Blood entering the LOV was probably shunted through the connecting uterine veins and possibly the ipsilateral ovarian vein as shown in Figure 3 [9]. Indeed, on more inferior axial slices, the left uterine veins appeared mildly congested and showed enhancement; however, this may be due to normal anterograde venous flow. The source of LRV obstruction was due to external compression by the PV and RRA and was seen on CT multiplanar reconstructions. Further imaging such as MRI or angiography would have added considerably to the results; however, they were not performed as there was no clinical justification.

NCP involving the RRA has been reported twice previously, although neither involved the PV. The authors of the first case observed a ventral takeoff of the RRA [6] whilst the authors of the second case noted a precipitously inferior course of the RRA being the driving factor [7]. We identified neither of the above observations in our case and suggest that a “tight” posterior course of the PV is the main cause to the left renal vein compression seen in our patient.

Whilst NCP is untreated due to its benign nature, a wide variety of operative and nonoperative treatments have been employed to treat NCS. If patients are younger than 18 years, conservative management with a 2-year-old observational period is considered best practice as up to 75% of symptomatic patients in this demographic will have complete resolution of their symptoms [2]. There is weak evidence to support angiotensin converting enzyme inhibitors and aspirin as part of nonoperative therapy, although larger studies are needed to confirm efficacy [2, 3]. Common operative approaches include LRV transposition ± Dacron wedge insertion, LRV bypass, medial nephropexy, and more recently extra-/intraluminal LRV stenting [2, 6]. Larger operations such as renal autotransplant, SMA transposition, and nephrectomy tend to be less efficacious although no large trials exist [2].

A more complete understanding of the possible pathologic anatomy that may underlie NCP/NCS is imperative to aid treating teams in diagnosis and management. NCP/NCS due to compression from the PV and the RRA has not been previously reported, making our case an important addition to the understanding of these conditions.
